# Supplementary material for: The influence of fluctuating population densities on evolutionary dynamics
Source: Evolution. 2019 Jun 11;73(7):1341–55. doi: 10.1111/evo.13756 (PMC6771508; doi:10.1111/evo.13756)
Supplement: Supplementary file 1 — Supporting Information [file EVO-73-1341-s001.zip › evo13756-sup-0002-SuppMat.pdf]

The influence of fluctuating population densities on evolutionary dynamics

Hanja Pisa,
Joachim Hermisson, Jitka Polechová

Evolution, 2019

Model: Gene flow between two divergent habitats

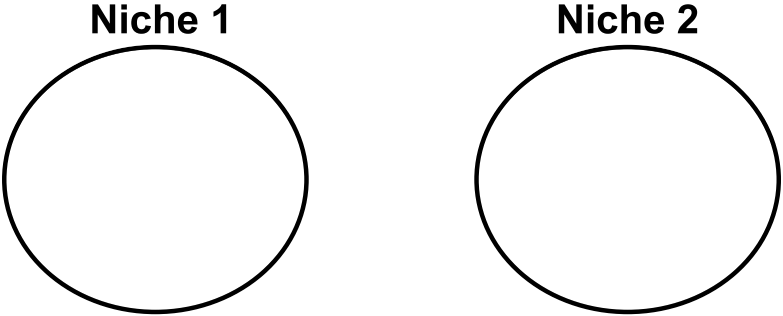

Model: Gene flow between two divergent habitats

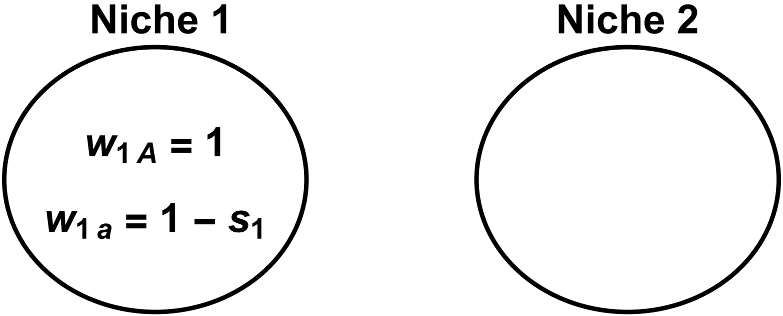

Alleles *A* and *a* (one locus, haploids)

Model: Gene flow between two divergent habitats

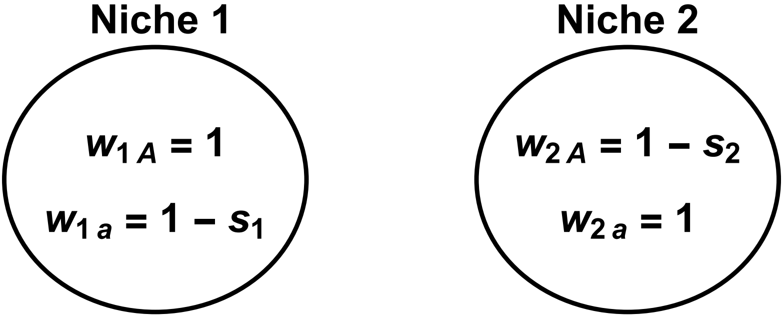

Alleles *A* and *a* (one locus, haploids)

Model: Gene flow between two divergent habitats

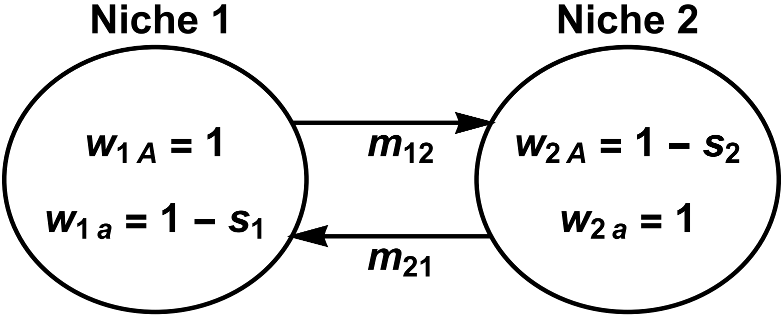

Alleles A and a (one locus, haploids)

Model: Gene flow between two divergent habitats

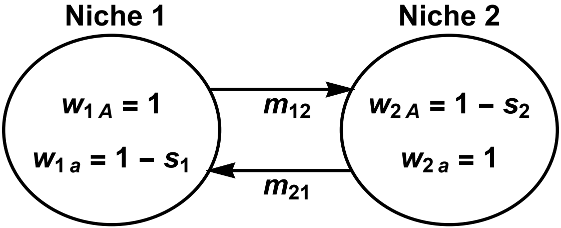

Model: Gene flow between two divergent habitats

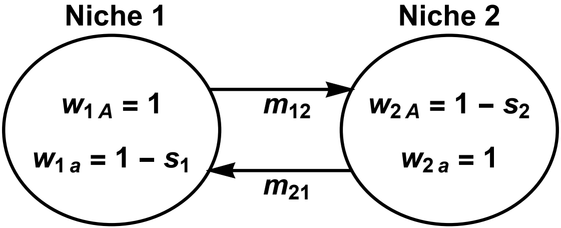

$p_i(t+1) = p'_i(t) \cdot \frac{w_{iA}}{\overline{w}'_i(t)}$

Model: Gene flow between two divergent habitats

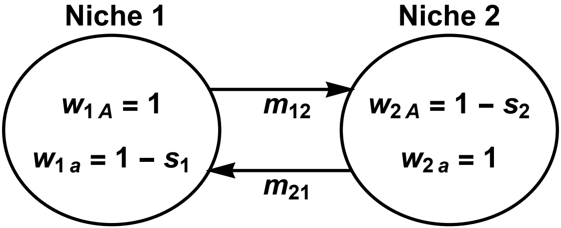

$$p_i(t+1) = p'_i(t) \cdot \frac{w_{iA}}{\overline{w}'_i(t)}$$

$$N_i(t+1) = N'_i(t) \cdot e^{r_i\left(1 - \frac{N'_i(t)}{K_i}\right)}$$

Model: Gene flow between two divergent habitats

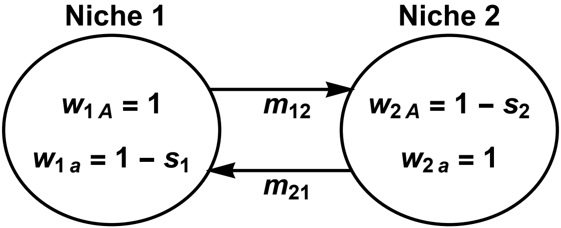

$$p_i(t+1) = p_i'(t) \cdot \frac{w_{iA}}{\overline{w}_i'(t)}$$

$$N_i(t+1) = N_i'(t) \cdot e^{r_i\left(1-\frac{N_i'(t)}{K_i}\right)\cdot \overline{w}_i'(t)}$$

Model: Gene flow between two divergent habitats

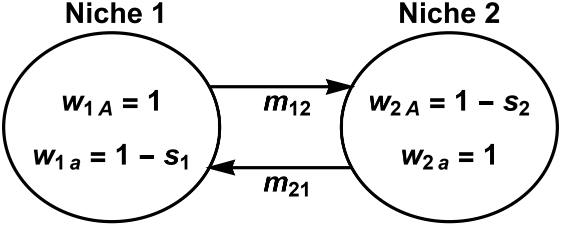

$$p_i(t+1) = p_i'(t) \cdot \frac{w_{iA}}{\overline{w}_i'(t)}$$

$$N_i(t+1) = N_i'(t) \cdot e^{r_i\left(1 - \frac{N_i'(t)}{K_i}\right) \cdot \overline{w}_i'(t)}$$

$$N_1'(t) = (1 - m_{12})N_1(t) + m_{21}N_2(t)$$
$$p_1'(t) = \frac{(1-m_{\mathbf{12}})p_{\mathbf{1}}(t)N_{\mathbf{1}}(t) + m_{\mathbf{21}}p_{\mathbf{2}}(t)N_{\mathbf{2}}(t)}{N_{\mathbf{1}}'(t)}$$
$$\overline{w}_i'(t) = p_i'(t)w_{iA} + (1 - p_i'(t))w_{ia}$$

Ecological dynamics change evolutionary prediction

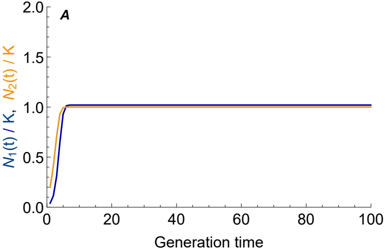

$r_1 = 1$
$r_2 = 1$

Ecological dynamics change evolutionary prediction

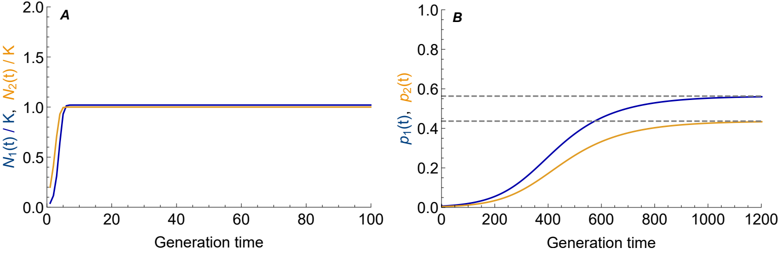

$r_1 = 1$

$r_2 = 1$, $s_1 = s_2 = 0.05$, $m_{12} = m_{21} = 0.1$

Ecological dynamics change evolutionary prediction

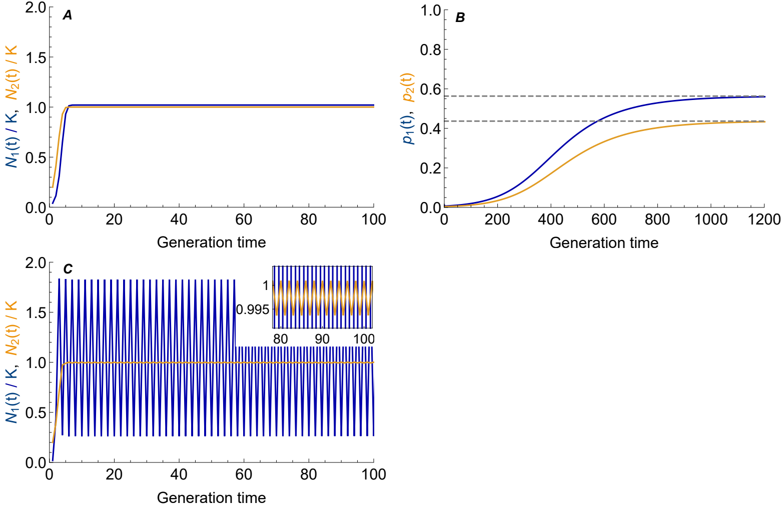

$r_1 = 1$ (A, B) and $r_1 = 2.5$ (C, D)
$r_2 = 1$, $s_1 = s_2 = 0.05$, $m_{12} = m_{21} = 0.1$

Ecological dynamics change evolutionary prediction

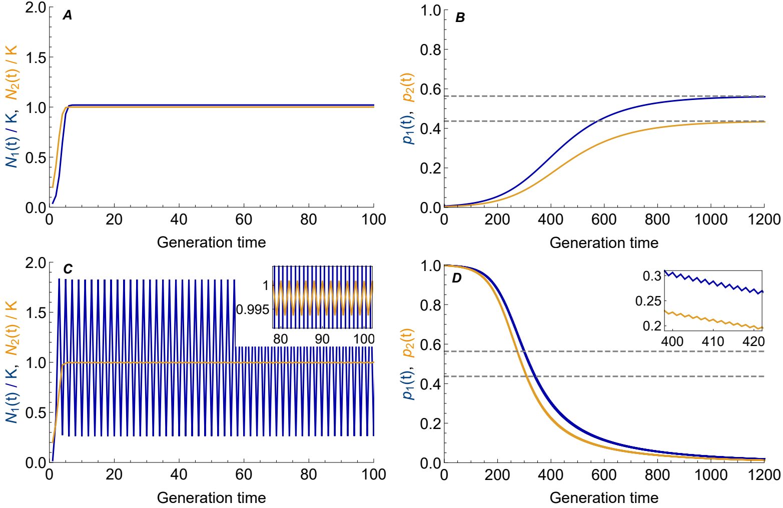

$r_1 = 1$ (A, B) and $r_1 = 2.5$ (C, D)

$r_2 = 1$, $s_1 = s_2 = 0.05$, $m_{12} = m_{21} = 0.1$

Stronger fluctuations correspond to lower equilibrium frequency of the locally adapted type

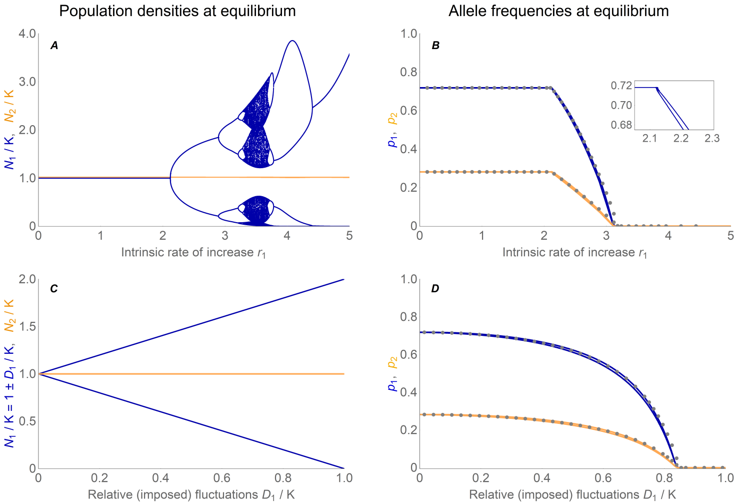

Mean backward migration increases significantly as density fluctuations grow

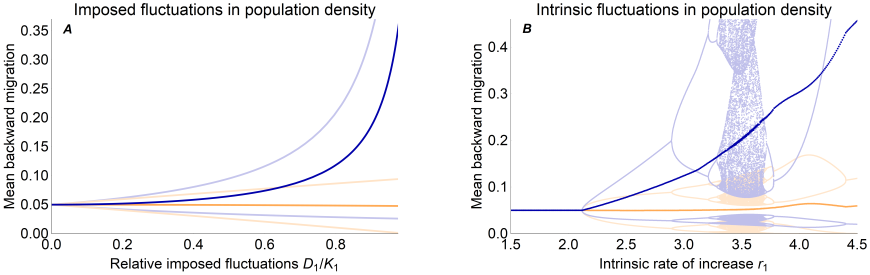

Mean backward migration approximates density fluctuations well (grey dots)

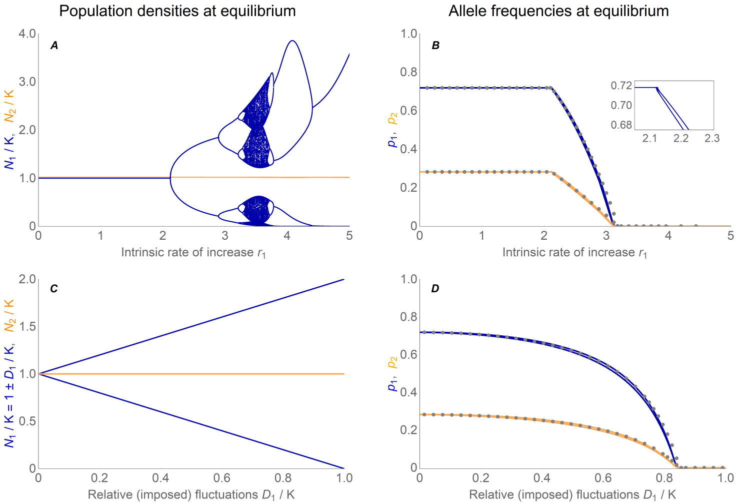

Stability of polymorphism under stable population sizes

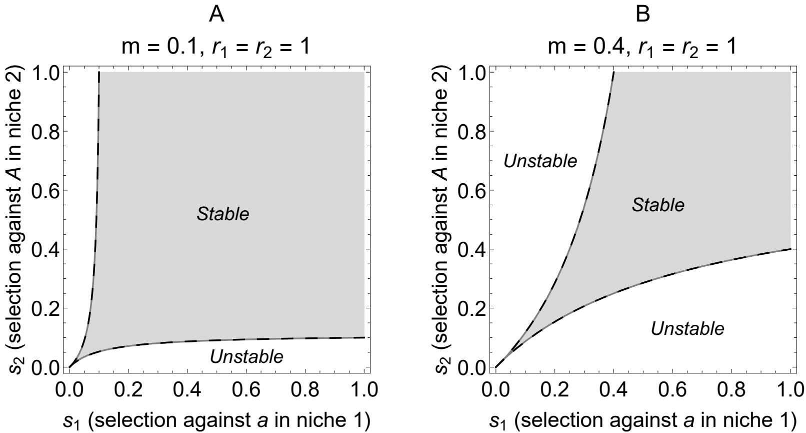

$$m \cdot \left| \frac{1}{s_1} - \frac{1}{s_2} \right| \; < \; 1 - m$$

Imposed fluctuations: stability of polymorphism

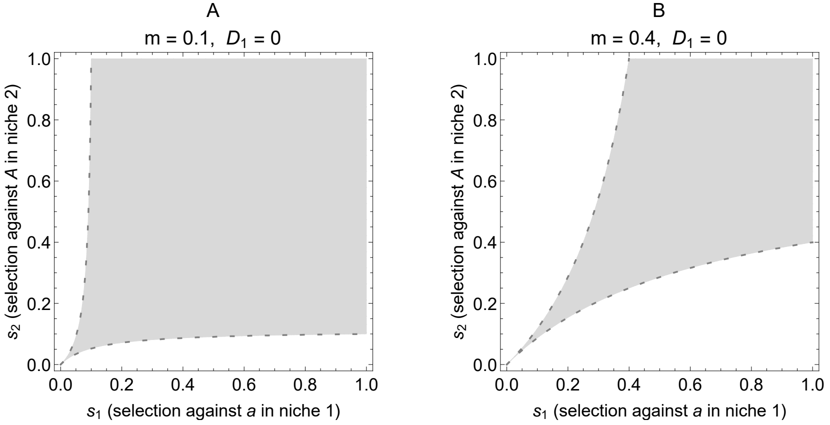

→ Stability of polymorphism for **stable** population densities

Imposed fluctuations: stability of polymorphism

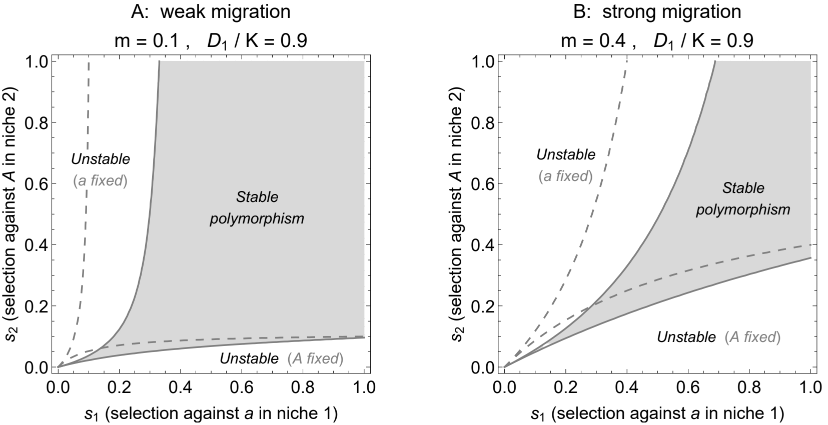

$\rightarrow$ Stability of polymorphism for **fluctuating** population densities

Imposed fluctuations in a continent-island model

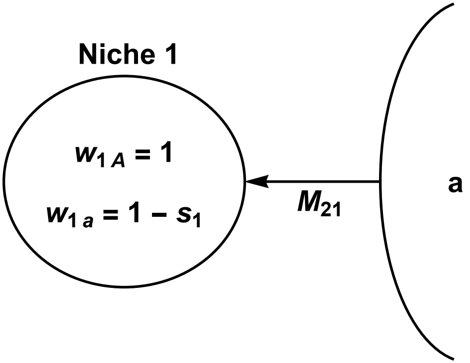

$N_1(t) = K_1 + D_1$
$N_1(t+1) = K_1 - D_1$

Imposed fluctuations, unidirectional migration

Resident type $\boldsymbol{A}$ survives on the island if and only if

$$\frac{D_1}{K_1} < \sqrt{\frac{1}{(2-s_1)s_1} - \left(\frac{1}{(2-s_1)s_1} - 1\right) \cdot \left(1 + \frac{M_{21}}{K_1}\right)^2}$$

Imposed fluctuations, unidirectional migration

Conditions for maintenance of polymorphism

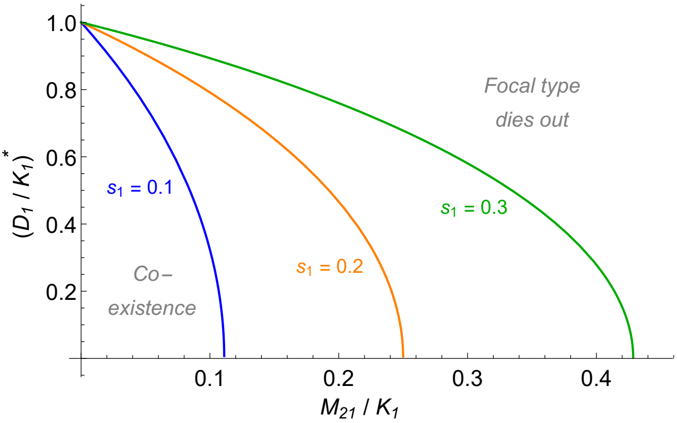

$$\left(\frac{D_1}{K_1}\right)^* := \sqrt{\frac{1}{(2-s_1)s_1} - \left(\frac{1}{(2-s_1)s_1} - 1\right) \cdot \left(1 + \frac{M_{21}}{K_1}\right)^2}$$

Imposed fluctuations, unidirectional migration

Conditions for maintenance of polymorphism

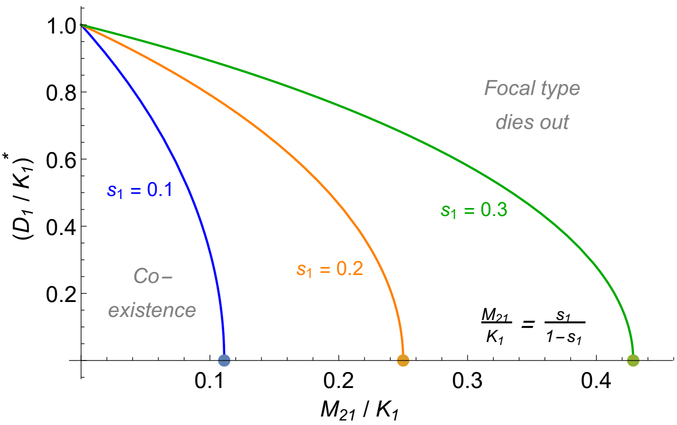

$$\left(\frac{D_1}{K_1}\right)^* := \sqrt{\frac{1}{(2-s_1)s_1} - \left(\frac{1}{(2-s_1)s_1} - 1\right) \cdot \left(1 + \frac{M_{21}}{K_1}\right)^2}$$

Imposed fluctuations, unidirectional migration

Conditions for maintenance of polymorphism

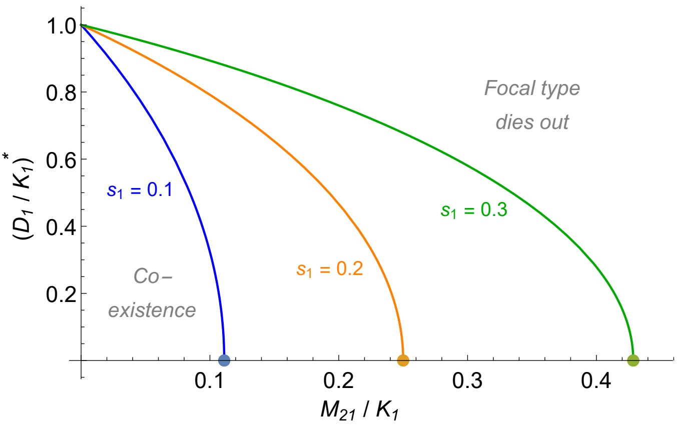

$$\left(\frac{D_1}{K_1}\right)^* := \sqrt{\frac{1}{(2-s_1)s_1} - \left(\frac{1}{(2-s_1)s_1} - 1\right) \cdot \left(1 + \frac{M_{21}}{K_1}\right)^2}$$

Imposed fluctuations, symmetric migration

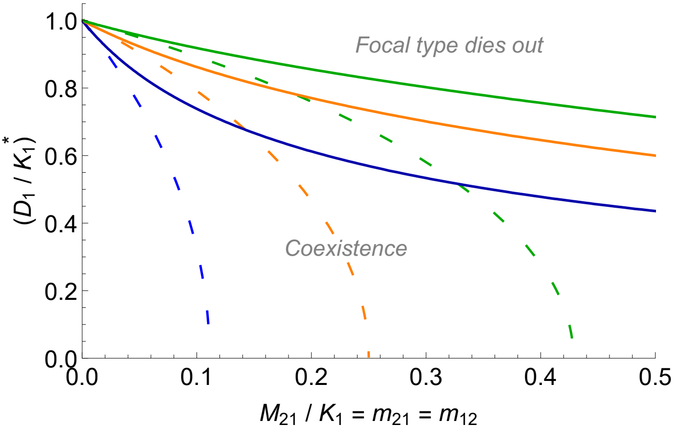

$s_1 = s_2 = 0.1, 0.2, 0.3$
