# Supplementary figures and images for: The influence of fluctuating population densities on evolutionary dynamics
Source: Evolution. 2019 Jun 11;73(7):1341–55. doi: 10.1111/evo.13756 (PMC6771508; doi:10.1111/evo.13756)

A

$$m = 0.1, r_1 = r_2 = 1$$

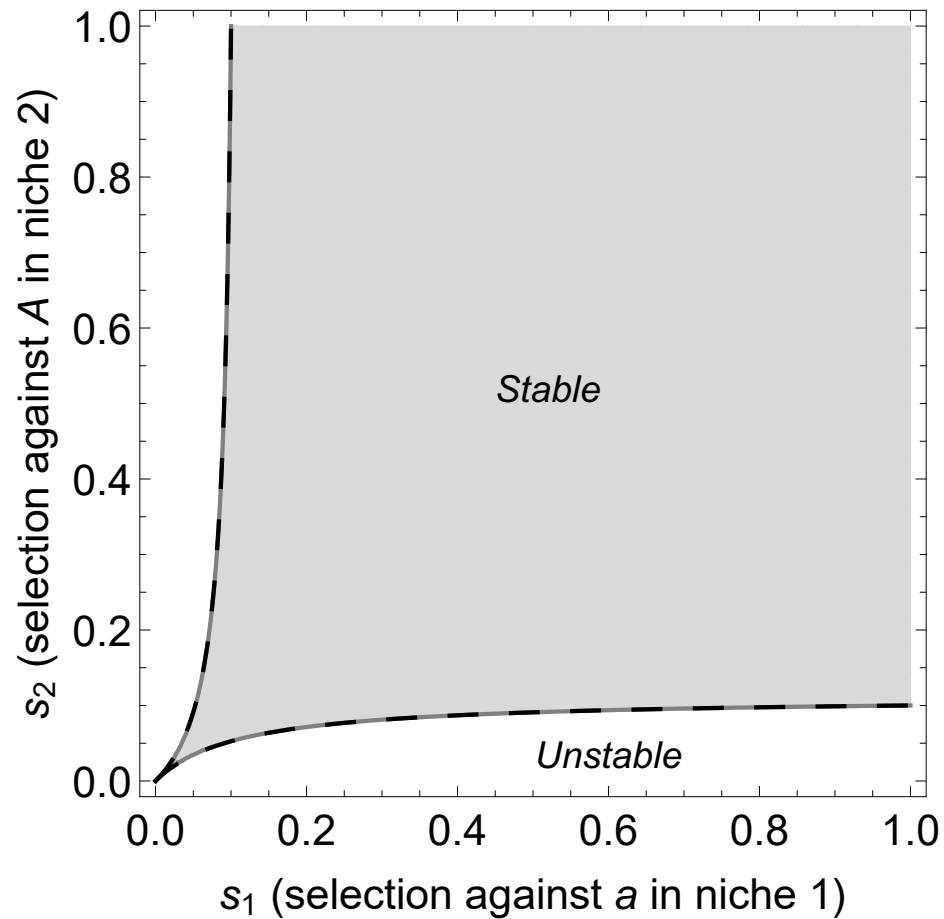

B

$$m = 0.4, r_1 = r_2 = 1$$

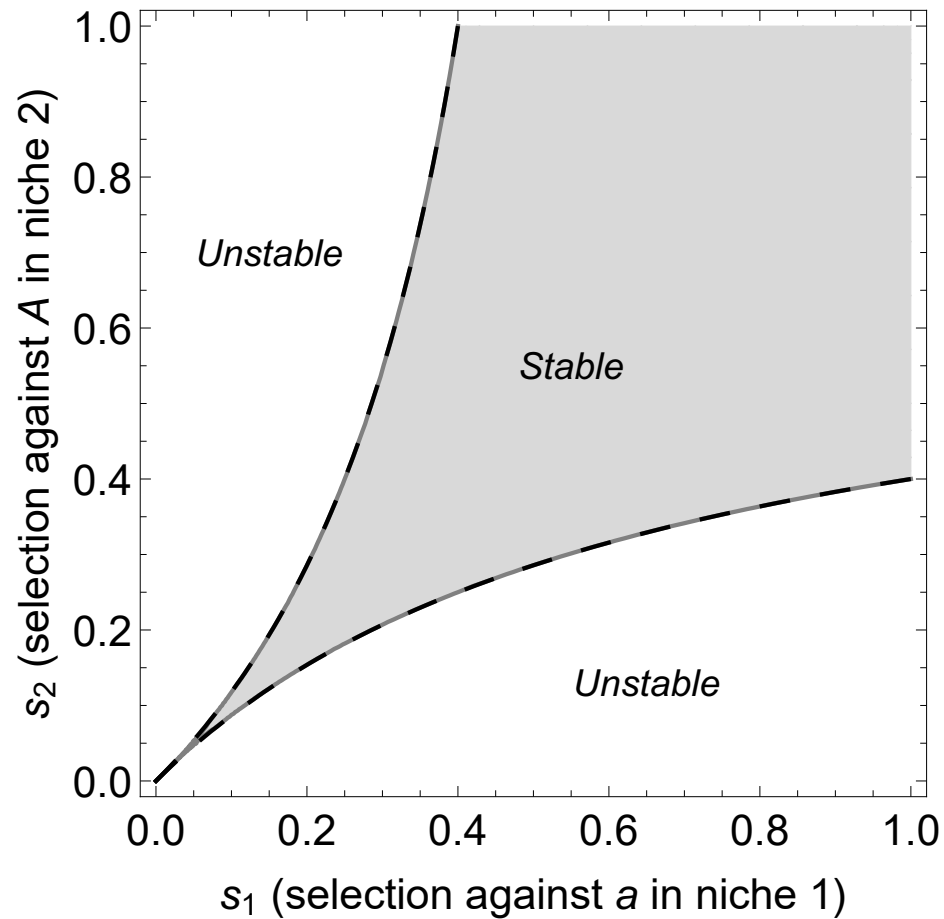

Supplement: Supplementary file 1 — Supporting Information [file EVO-73-1341-s001.zip › evo13756-sup-0003-FigureS1.pdf]

$$K_1 = N_2 = 100$$

$$D_1 = 50$$

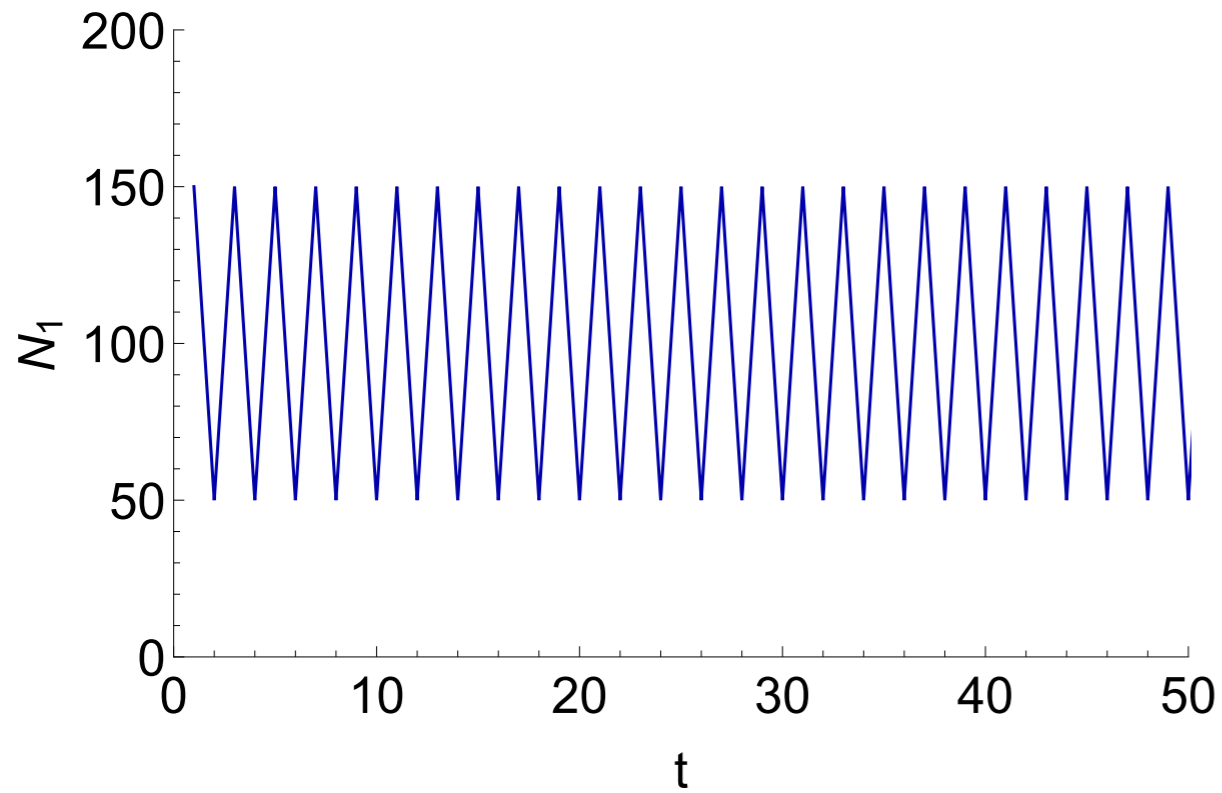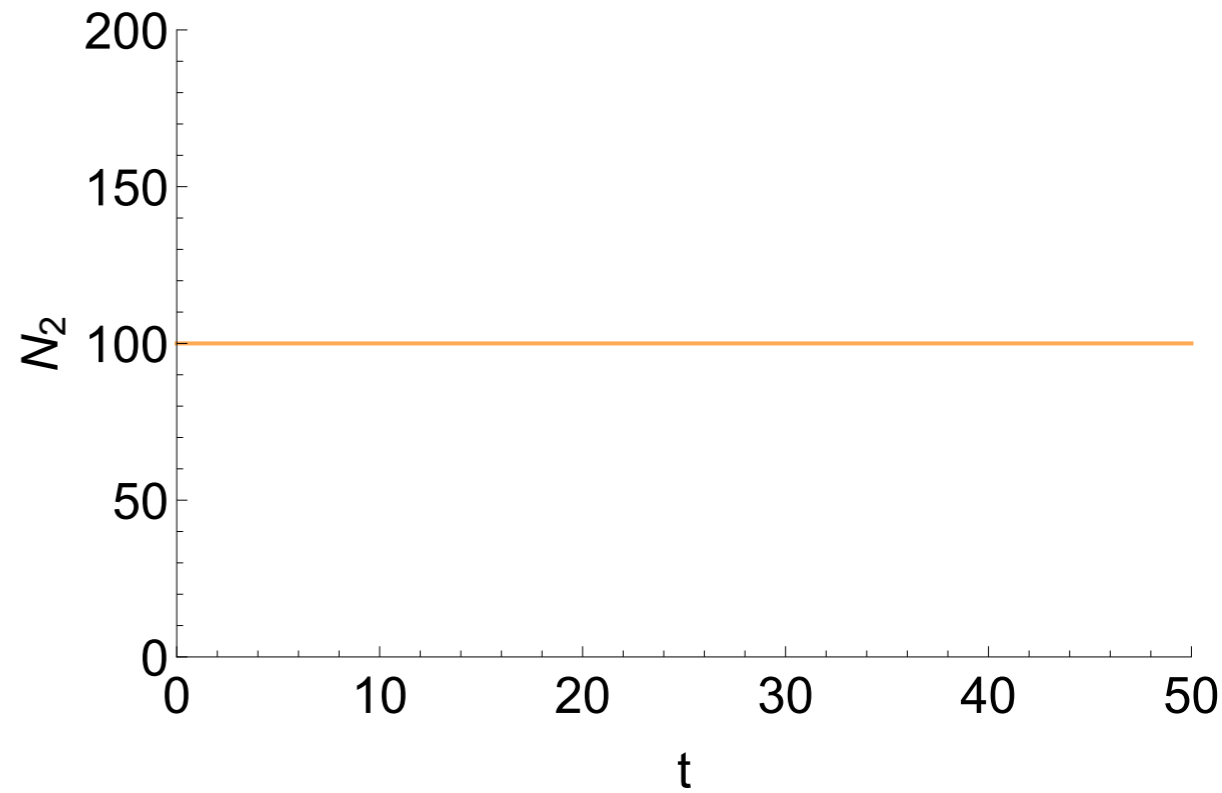

Supplement: Supplementary file 1 — Supporting Information [file EVO-73-1341-s001.zip › evo13756-sup-0004-FigureS1.pdf]

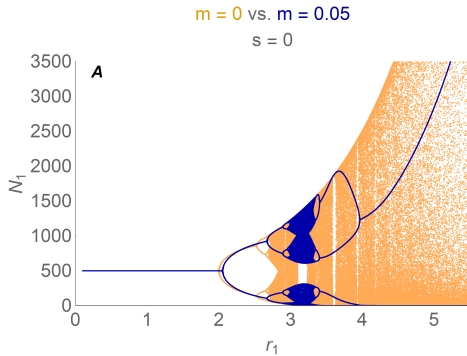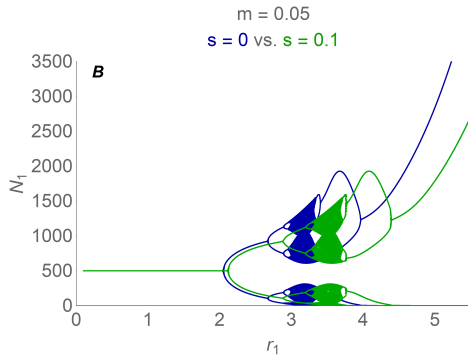

Supplement: Supplementary file 1 — Supporting Information [file EVO-73-1341-s001.zip › evo13756-sup-0005-FigureS1.pdf]

A

 $m = 0, s = 0$ 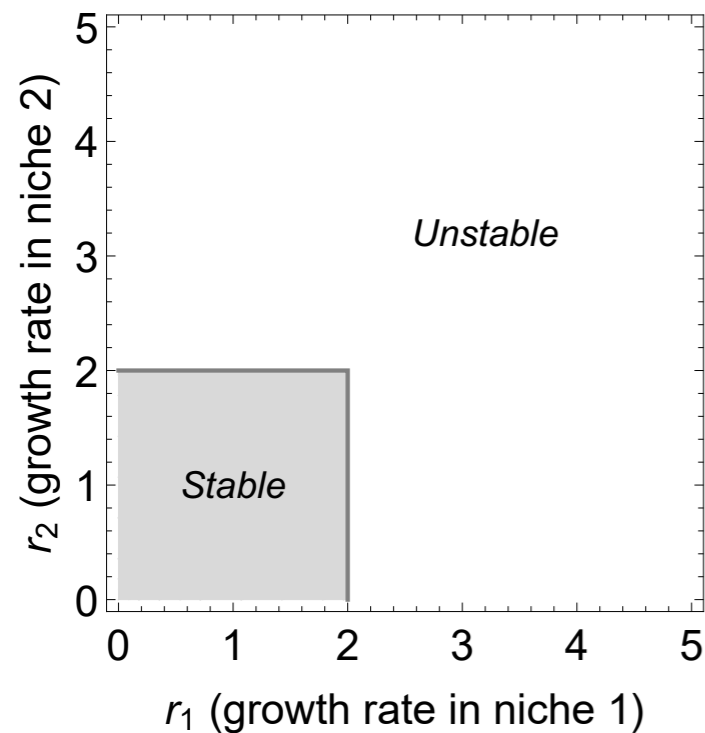

B

 $m = 0.4, s = 0$ 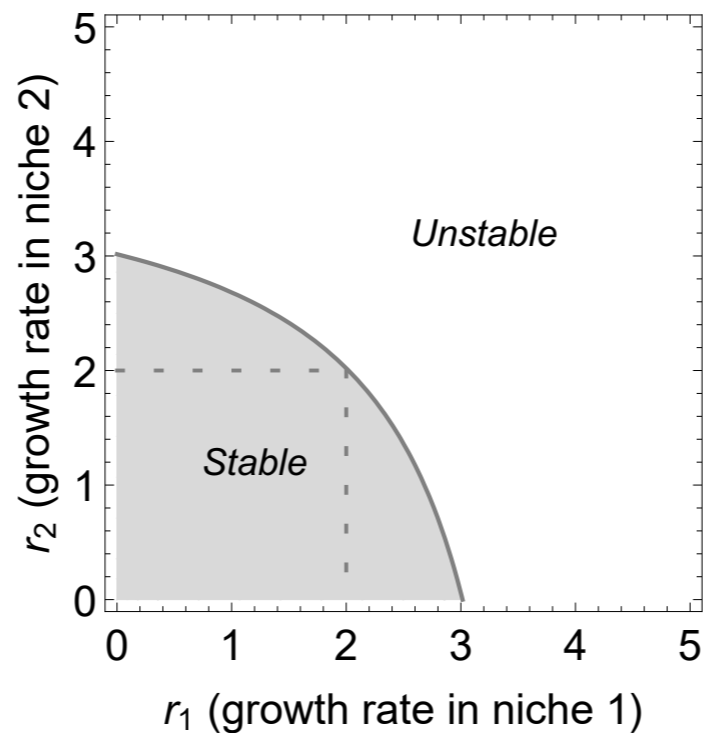

C

 $m = 0.4, s = 0.3$ 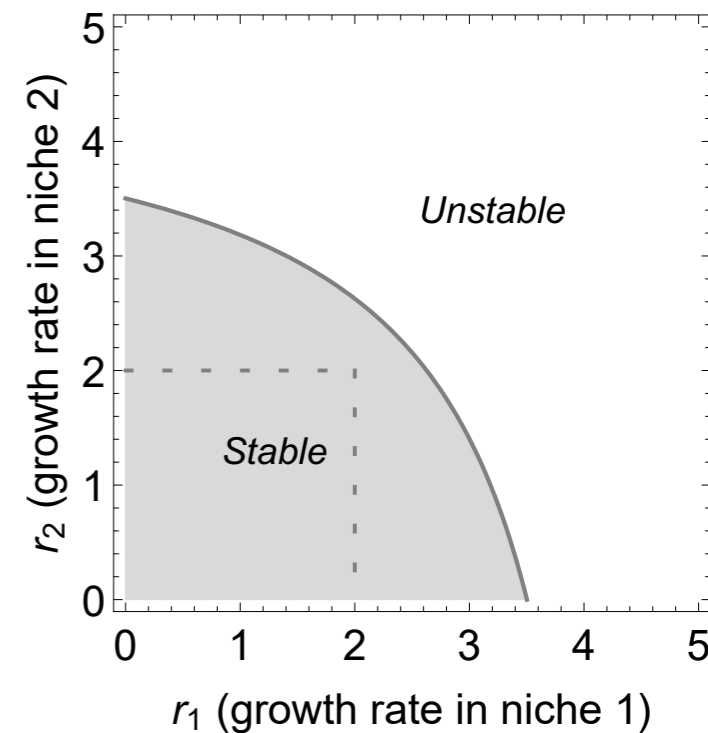

D

 $m = 0.4, r_2 = 1$ 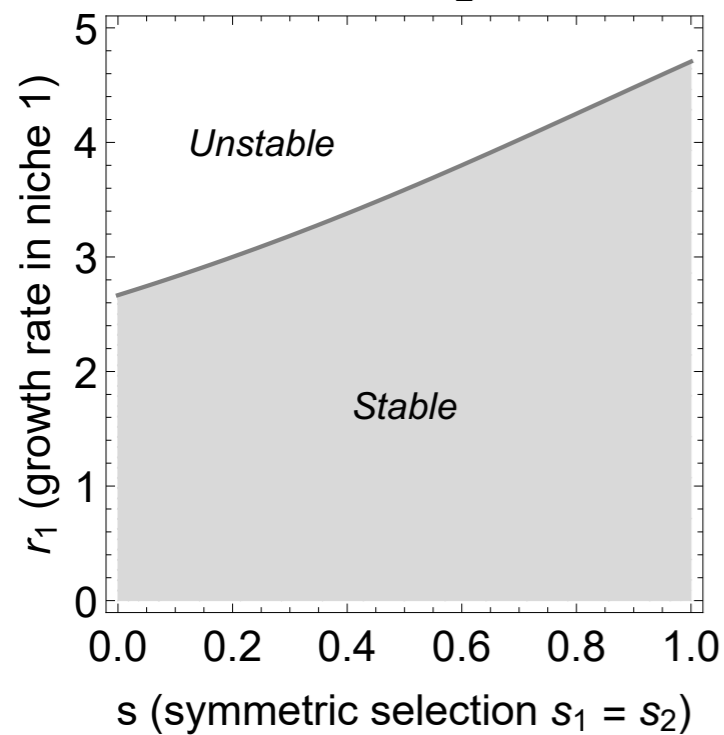

E

 $m = 0.4, s_2 = 0.3, r_2 = 1$ 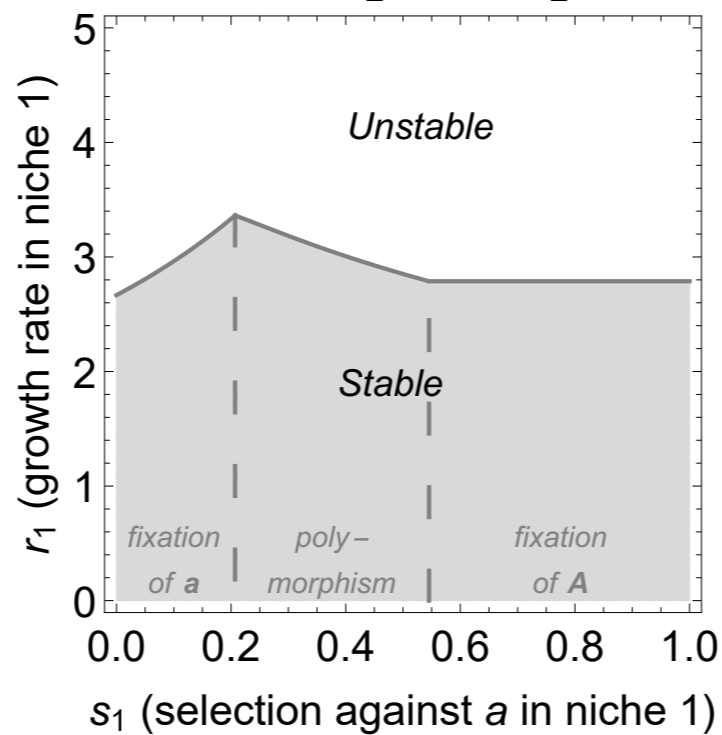

F

 $m = 0.4, s_1 = 0.3, r_2 = 1$ 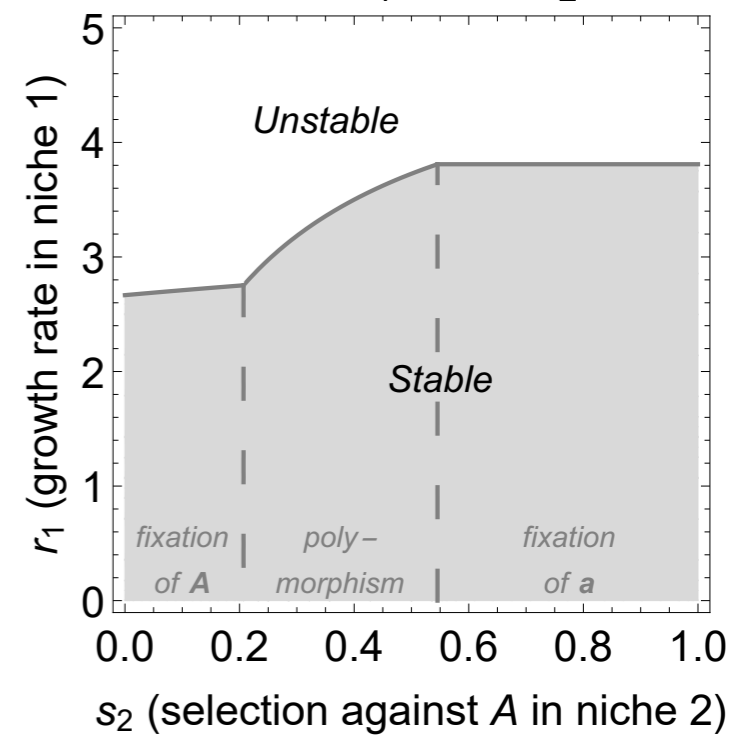

Supplement: Supplementary file 1 — Supporting Information [file EVO-73-1341-s001.zip › evo13756-sup-0006-FigureS1.pdf]
